# Biodegradation of Organophosphorus Compounds Predicted by Enzymatic Process Using Molecular Modelling and Observed in Soil Samples Through Analytical Techniques and Microbiological Analysis: A Comparison

**DOI:** 10.3390/molecules25010058

**Published:** 2019-12-23

**Authors:** Monique Cardozo, Joyce S. F. D. de Almeida, Samir F. de A. Cavalcante, Jacqueline R. S. Salgado, Arlan S. Gonçalves, Tanos C. C. França, Kamil Kuca, Humberto R. Bizzo

**Affiliations:** 1 Natural Products Research Institute (IPPN), Federal University of Rio de Janeiro (UFRJ), CCS, Bloco H, Cidade Universitária, Rio de Janeiro 21941-902, Brazil; samirfac@yahoo.com.br (S.F.d.A.C.); humberto.bizzo@embrapa.br (H.R.B.); 2 Institute of CBRN Defense (IDQBRN), Avenida das Américas 28705, Rio de Janeiro 23020-470, Brazil; capjrsoares@gmail.com; 3 Laboratory of Molecular Modeling Applied to Chemical and Biological Defense (LMACBD) Military Institute of Engineering (IME), Praça General Tibúrcio 80, Rio de Janeiro 22290-270, Brazil; joycesfdalmeida@gmail.com (J.S.F.D.d.A.); tanosfranca@gmail.com (T.C.C.F.); 4 Department of Chemistry, Faculty of Science, University of Hradec Kralove, 50003 Hradec Kralove, Czech Republic; 5 Federal Institute of Education, Science and Technology, Avenida Ministro Salgado Filho, 1000, Soteco, Vila Velha 29106-010, Espírito Santo, Brazil; arlansgoncalves@gmail.com; 6 Federal University of Espirito Santo- Unit Goiabeiras, Vitória 29075-910, Espírito Santo, Brazil; 7 Embrapa Agroindústria de Alimentos, Avenida das Américas 29501, Rio de Janeiro 23020-470, Brazil

**Keywords:** bioremediation, phosphotriesterase, organophosphorus compounds, molecular modeling

## Abstract

Organophosphorus compounds (OP) are chemicals widely used as pesticides in different applications such as agriculture and public health (vector control), and some of the highly toxic forms have been used as chemical weapons. After application of OPs in an environment, they persist for a period, suffering a degradation process where the biotic factors are considered the most relevant forms. However, to date, the biodegradation of OP compounds is not well understood. There are a plenty of structure-based biodegradation estimation methods, but none of them consider enzymatic interaction in predicting and better comprehending the differences in the fate of OPs in the environment. It is well known that enzymatic processes are the most relevant processes in biodegradation, and that hydrolysis is the main pathway in the natural elimination of OPs in soil samples. Due to this, we carried out theoretical studies in order to investigate the interactions of these OPs with a chosen enzyme—the phosphotriesterase. This one is characteristic of some soils’ microorganisms, and has been identified as a key player in many biodegradation processes, thanks to its capability for fast hydrolyzing of different OPs. In parallel, we conducted an experiment using native soil in two conditions, sterilized and not sterilized, spiked with specific amounts of two OPs with similar structure—paraoxon-ethyl (PXN) and O-(4-nitrophenyl) O-ethyl methylphosphonate (NEMP). The amount of OP present in the samples and the appearance of characteristic hydrolysis products were periodically monitored for 40 days using analytical techniques. Moreover, the number of microorganisms present was obtained with plate cell count. Our theoretical results were similar to what was achieved in experimental analysis. Parameters calculated by enzymatic hydrolysis were better for PXN than for NEMP. In soil, PXN suffered a faster hydrolysis than NEMP, and the cell count for PXN was higher than for NEMP, highlighting the higher microbiological toxicity of the latter. All these results pointed out that theoretical study can offer a better comprehension of the possible mechanisms involved in real biodegradation processes, showing potential in exploring how biodegradation of OPs relates with enzymatic interactions.

## 1. Introduction

Nowadays, the most widely used pesticides belong to the organophosphorus (OPs) group. These compounds are used to control agricultural and household pests, corresponding to 38% of the total pesticides used globally [1]. Intoxication by OPs remains a threat to human health, being responsible for 3 million poisonings and 200,000 deaths annually [2].

The toxicity of these compounds is due to the inhibition of the enzyme acetylcholinesterase (AChE; EC 3.1.1.7), leading to the accumulation of the neurotransmitter acetylcholine (ACh) and subsequent over-activation of cholinergic receptors in many parts of the body [3]. These compounds have been implicated in several nerve and muscular diseases in human beings. The acute adverse effects include miosis, bronchorrhea, bronchoconstriction, bradycardia, emesis, skeletal muscle contraction, tachycardia, seizures, respiratory arrest, and other symptoms, leading to death [4].

Moreover, the commodities and places of contamination are also worrying. The wide large-scale use of OPs causes serious concerns in terms of food safety and environmental pollution [5]. OPs can be found in meat and milk, as many of them are fat-soluble and are used directly on animal skin, and are found also in agricultural commodities such as grains, vegetables, and fruits, mainly due to the effects of bioaccumulation [1,6]. For vector control, especially to prevent mosquito proliferation such as the species *Aedes aegypti* L., OPs are among the most effective known methods due to their larvicide and adulticide action. For this reason, OPs are intensively used in tropical regions to prevent the transmission of illnesses such as dengue, Zika, yellow fever, and chikungunya [7,8]. However, it is estimated that only 5% of this kind of compound reaches the targeted organisms [9], and the remainder may cause significant changes in ecological parameters.

In addition, OPs with high neurotoxic effects can also be used as chemical warfare agents. Despite the Chemical Weapons Convention (CWC) and the joint effort of state parties to eradicate the danger posed by CW, recent events have demonstrated that it may continue to be employed in many ways in the form of state-sponsored programs, actions of terrorists, or other criminal groups, and even by individual use [10]. Events such as the confirmation of neurotoxic agents being used in the Syria conflicts and the murder of the North Korean leader’s stepbrother via a high dose of Ethyl ({2-[bis(propan-2-yl)amino]ethyl}sulfanyl)(methyl)phosphinate (VX) in 2017, illustrated that the risk is high and is increasing [11]. Even in the official world remaining stockpile, it is estimated that there are 2105 metric tons of extremely toxic OPs, including sarin, soman, and VX [12], a perpetual an environmental risk with the possibility of soil and water contamination due to the deterioration of CW and the impact of destruction operations [13].

It is well known that natural microbiota interferes in the fate of OPs in the environment. The microorganism-mediated transformation or degradation of contaminants into nonhazardous or less-hazardous substances is called bioremediation [14]. The use of various organisms such as bacteria, fungi, algae, and plants as efficient agents has been reported. The process needs to be better understood, as it is dependent on many factors. However, the enzymatic interactions are considered the most relevant ones, thanks to their capability to catalyze the degradation process using different substrates [15].

The soil is the richest habitat in microbial diversity, estimated to contain about 10 billion microorganisms per gram. Soil microorganisms and microbiomes have been studied in a variety of conditions that have allowed not only a better understanding of the microbial composition and functioning in the environment, but have also provided important tools for searching for new molecules, such as enzymes and metabolites, for potential biotechnological use, such as in biocatalysts and antibiotics [16].

Considering the fact that toxic OPs are not natural products, the bacterial enzymes seemingly have evolved, within a relatively short period, to specifically hydrolyze associated functional groups [17]. The microbial cells, with the action of some enzymes, present detoxification activities for bioremediation purposes. If a nerve agent has been used, OP-degradation enzymes have the potential to promote a fast hydrolysis compared to traditional chemical methods, with less impact to the environment [9].

The way in which the microorganism’s soil community metabolizes OPs is still under investigation. Some pathways in both Gram-positive and Gram-negative bacteria under aerobic conditions are understood, and the general tendency is that degradation models for most OPs tend to share the same characteristics, with hydrolytic enzymes playing a fundamental role in the process [18].

It has been reported in soil treated with OPs a rising activity of a variety of microorganisms’ enzymes capable of detoxifying OPs through hydrolysis, such as, acid and alkaline phosphatase, phosphodiesterase, phosphotriesterase (PTE) and dehydrogenase [19,20]. Among them, enzymes listed in the category of phosphoric triester hydrolases have special importance in that they work over a wide temperature and pH range, and are capable of degrading many OP substrates, acting in the metabolism of compounds such as nerve agents and pesticides [21,22]. These enzymes were further broken down into two subgroups —the aryldialkylphosphatases (EC 3.1.8.1) (also referred to as organophosphorus hydrolases and PTEs) that prefer substrates bearing P–O or, alternatively, P–S bonds; and the diisopropyl-fluorophosphatases (EC 3.1.8.2) (also including organophosphorus acid anhydrolase (OPAA)), which are more active against OP compounds with P–F or P–CN bonds [21].

PTE is encoded by the organophosphorus-degrading (opd) gene and this is part of the amidohydrolase superfamily [9,21]. All PTEs are metal-dependent hydrolases, that is, there is a requirement for a divalent metal, which directly binds to the substrate to favor the catalysis process [23]. Zinc was found to be the native metal; however, other bivalent cations, such as Cd^2+^ and Co^2+^, can also support activity [24,25]. The evaluation of kinetic constants obtained for paraoxon (PXN) by metal-substituted PTEs, including Zn/Zn, Co/Co, Cd/Cd, Cd/Co, and Cd/Zn, shows that the Cd/Cd PTE has the highest K_m_ and pKa values and the lowest k_cat_/K_m_ correlation [24].

PTE presents a strong hydrolysis for a range of insecticides such as phosphothioesters and phosphorofluoridates, including chemical weapons such as sarin and soman, and is the only enzyme capable of hydrolyzing P–S bonds in OPs [22]. The best well-characterized PTE comes from *Brevundimonas diminuta*, and is a homodimer (35 kDa per monomer), with the overall folding pattern consisting of an α/β barrel with eight strands of a parallel β-pleated sheet. The two divalent metal ions are situated at the C-terminal portion of the TIM (triosephosphate isomerase) barrel motif [26]. A histidine-rich region (residues His55, His57, His201, His230, Asp301, and Lys169) facilitates binding of the metals in the active site. Lys169, which is carboxylated, and a water molecule (or hydroxide ion) serve as bridging ligands between two metal cations, which are essential for nucleophilic attack to the phosphorus center of the OPs [21].

It can be affirmed that biodegradation of OPs is an important process to protect living beings and the environment of the hazardous effects of this compound. As the hydrolysis is the principal process involved, the degradation will be better comprehended through the study of the behavior of hydrolytic enzymes in the media [9]. It is known that there are different ways of estimating biodegradation, with most of them being structure-based methods [27]. However these models are limited. There is also a search for ways and models to predict and analyze the natural behavior of different compounds. It is a consensus that the most of the degradation process follows the first-order kinetics but defining the set of parameters that can influence most in the observed degradation pattern is a challenge that, in general, demands a huge experimental effort [28,29].

**Figure 1:**
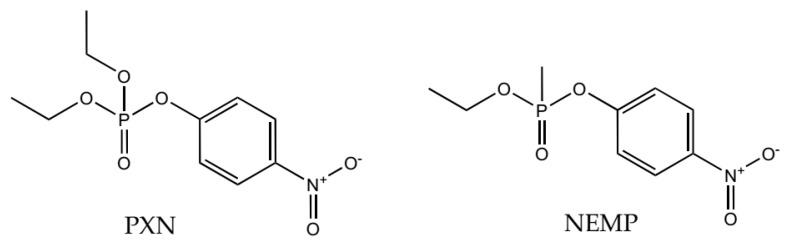
Structures of paraoxon-ethyl (PXN) and *O*-(4-nitrophenyl) *O*-ethyl methylphosphonate (NEMP).

**Figure 2:**
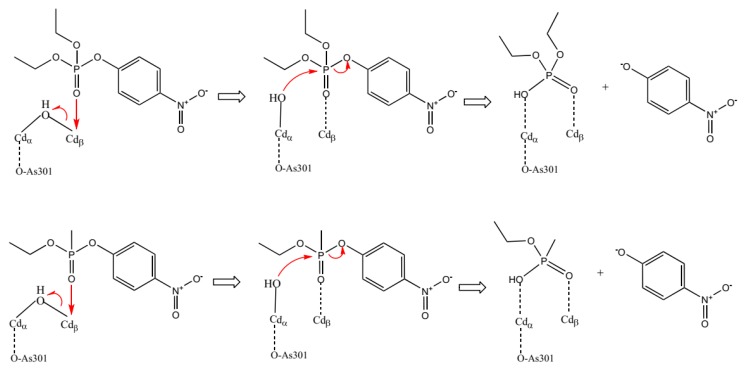
A proposed general pesticide-degrading mechanism for the phosphotriesterase (PTE)-catalyzed hydrolysis of PXN and NEMP. Adapted from Zhang et al. [26].

In order to investigate the degradation process, molecular modeling studies were performed in the present work to compare the stability and interactions of two OPs, a phosphate (PXN) and a phosphonate (NEMP), with similar structures (Figure 1) in the active site of PTE. The hydrolysis mechanism proposed is presented in Figure 2. After that, we evaluated the natural process of degradation of these OPs in a native soil matrix submitted to two different conditions: sterilized and non-sterilized. Using analytical techniques and statistic treatment of the data obtained, it was possible to quantify the reduction of these compounds in a period of time, as well as to monitor the formation of degradation products.

## 2. Results and Discussion

### 2.1. Molecular Docking Studies

**Figure 3:**
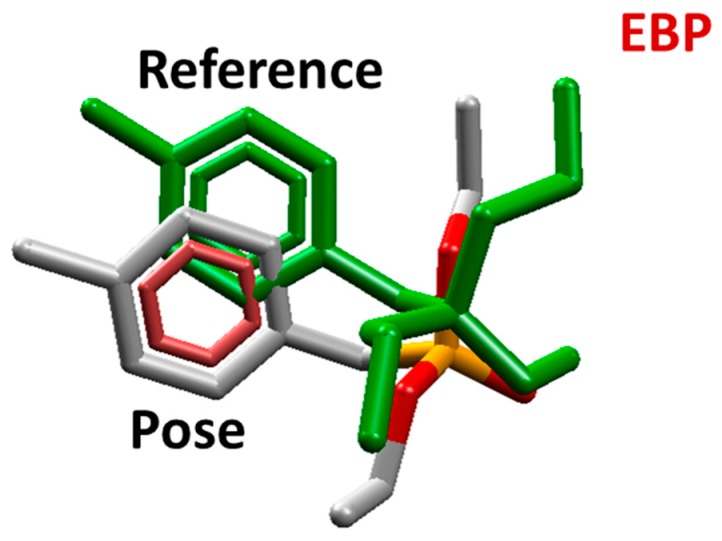
Best redocking pose. EBP: diethyl 4-methylbenzylphosphonate.

Molecular docking studies were performed to investigate the interactions of PXN and NEMP with the active site of PTE. The docking methodology was validated through a redocking procedure, using the reference ligand diethyl 4-methylbenzylphosphonate (EBP) inside the crystallographic structure of the enzyme. For the best conformation (pose), chosen according to atom coordinate superposition criteria, the root mean square deviation (RMSD) of 1.89 Å and intermolecular energy of −50.54 kcal/mol were obtained, satisfying validation requirements [30]. Figure 3 shows the superposition of the best pose obtained and the reference ligand.

**Figure 4:**
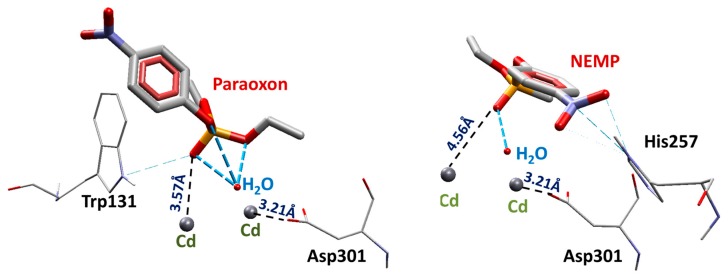
Best docking poses for PXN and NEMP.

After validation, molecular docking studies were performed for PXN and NEMP inside PTE. The results were analyzed considering the distance from the phosphorus double-bonded oxygen atom of OP to one of the Cd atoms (O(OP)-Cd), and also the distance from the sp^2^ oxygen atom of residue Asp301 to the other Cd atom (O(Asp301)-Cd), in order to verify whether the docking results could reproduce the configuration necessary for the occurrence of the proposed hydrolysis mechanism [26]. The best poses were also chosen according to intermolecular energy values. Table 1 presents the docking results for the best poses of each OP. The energy results suggest that PXN had higher affinity for the enzyme than NEMP, which may have favored the degradation. The hydrogen bond interaction (H-bond) with residue Trp131 contributed to the stabilization of PXN, and the H-bond with His257 contributed to the stabilization of NEMP in the active site of the enzyme. Figure 4 shows the best poses obtained for OP and NEMP.

### 2.2. Molecular Dynamics Study

**Figure 5:**
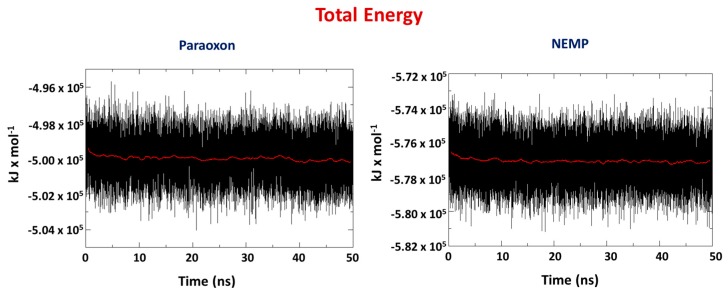
Total energy for the systems PTE/PXN and PTE/NEMP during 50 ns of molecular dynamics (MD) simulation.

Molecular dynamics (MD) simulations were performed with the best poses from docking studies to verify the behavior of both OPs during 50 ns. The total energy values for the systems PTE/OP are presented in Figure 5. The negative values suggest stabilization over time, with a lower average value for NEMP. For PXN, the average value obtained was −4.99 × 10^−5^ kJ/mol, whereas for NEMP it was −5.77 × 10^−5^ kJ/mol. Both systems stabilized from the beginning of the simulations.

**Figure 6:**
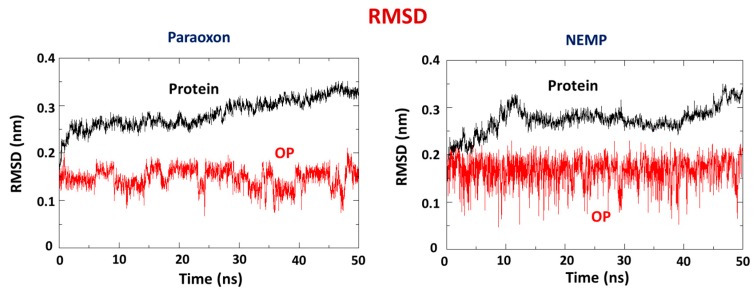
Root mean square deviation (RMSD) plots for PTE/PXN and PTE/NEMP during 50 ns of MD simulation.

RMSD plots PTE/PTE and ligand/ligand (Figure 6) were done to verify the stability of PTE and the OP during the MD simulation. As can be seen, both systems presented stabilization along the time corroborating previous energy results. In addition, PXN was more stable, with a lower RMSD and less fluctuation than NEMP. It corroborated docking results, suggesting a higher affinity of PXN for the enzyme. Additionally, the RMSD plots PTE/ligand were also done in order to verify the behavior of each ligand inside the protein. As shown in Figure S1 of Supplementary Information, PXN stabilized around 5–8 Å from the active site, whereas NEMP stabilized around 20 Å.

**Figure 7:**
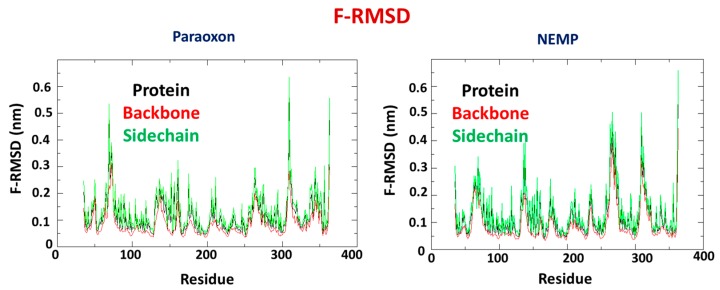
RMSD fluctuation (F-RMSD) plots for PTE/PXN and PTE/NEMP during 50 ns of MD simulation.

RMSD fluctuation (F-RMSD) studies for both systems were also performed to verify the enzyme regions that present higher RMSD. For both systems, the backbone presented lower fluctuation than the overall protein, which presented lower fluctuation than sidechains, as expected. However, it is important to notice that in the system PTE/PXN, the regions close to active site (residues His55, His57, Lys169, His201, Asp301, and His230) presented higher fluctuation than in the system PTE/NEMP, suggesting that PXN may affect these regions in a more significant way than NEMP, corroborating docking results and previous MD results. Figure 7 shows F-RMSD results for both systems.

**Figure 8:**
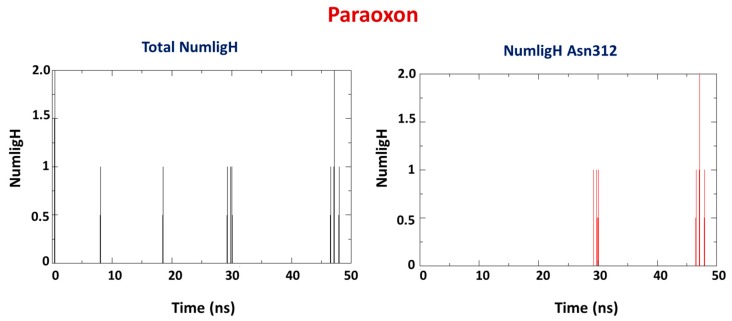
H-bond prevalence for the system PTE/PXN and PTE/NEMP.

**Figure 9:**
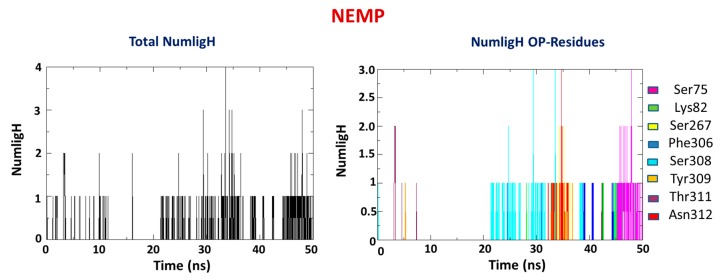
H-bond prevalence for the system PTE/NEMP.

Occurrence of H-bond interactions were also investigated for comparison with docking results, along with verification of which bonds remained during the simulation and the new ones formed. It is observable in Figure 8 and Figure 9 that PXN presented an average number of H-bonds lower than NEMP (Table 2). This meant that a higher number of residues contributed to the stabilization of NEMP than to PXN. Also, formation of the weak H-bonds with residues Trp131 and His257, as suggested by the docking studies, was not confirmed by the MD simulations. It is important to notice that NEMP presented, most of time, interaction with residues that were farther from the active site of PTE. This was probably a consequence of the average distance studies and its higher mobility inside the enzyme, as discussed below.

**Figure 10:**
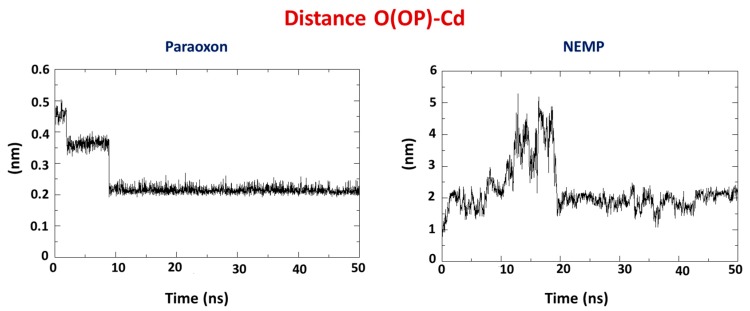
Average interatomic distance O(OP)-Cd for PXN and NEMP.

Average distances are a very important criteria to infer if the proposed hydrolysis mechanism [26] is suitable for PXN and NEMP degradation by PTE. Similarly to docking studies, the interatomic distances from the phosphorus double-bonded oxygen atom of the OP to one of the Cd atoms (O(OP)-Cd) were monitored during the 50 ns of MD simulations for both OPs in order to verify whether they kept the configuration favorable to the hydrolysis shown in Figure 2. Results (Figure 10) showed a great difference between them. Although PXN keeps very close to the Cd atom during all the simulation (around 2 Å), NEMP stabilized around 20 Å further than Cd. This suggested that the hydrolysis may have been the preferential degradation mechanism of PXN by PTE; however, it was maybe not the main mechanism for NEMP degradation. These results also corroborated docking studies that showed NEMP farther from the active site of the enzyme.

Experimental degradation studies in a soil sample with the presence of natural microorganisms were performed for PXN and NEMP. The results were analyzed and compared with theoretical results.

### 2.3. Total Cell Count and Recovering from Soil Sample

Microbial plate counts observed in non-sterile soil sample indicated that the soil microorganisms remained viable throughout the study in all types of samples, as shown in Table 3. The results were expressed in colony forming unit per gram of soil (CFU/g). The initial exposures doses were 40 micromole of PXN and NEMP per kilogram of soil (µmol/kg).

The addition of water at the same volume that solution was applied in other samples guaranteed the maintenance of humidity level in all soil samples, which is an important parameter in terms of microbiological activity. The cell count for natural soil showed that the number of cultivable forms of microorganisms remained virtually unchanged over time under the environment conditions analyzed. However, there was a slightly increasing pattern until 30 days, and a decreasing in the number of microorganisms after 40 days.

The cell counting results for soil samples with OP contamination in this study demonstrated that the presence of OPs in soil adversely affected soil microbes. The difference in the initial cell count between samples was significant with soil without contamination, having 14 times more cells then the soil with PXN and 90 times more cells then the soil with NEMP. These data are consistent with the toxicity of pesticides to the susceptible microbial species [31]. NEMP is considered more toxic than PXN because of the presence of the methyl group directly bound to phosphorous. This effect was shown in the cell count, which meant that less microorganisms were resistant to NEMP than to PXN.

After the initial effect, the tendency for the remaining soil microbes was to be adapted to the presence of OPs, and it was also possible that there was an increase in the biomass of the resistant forms because of the reduction of competition [31]. However, the results did not present an increasing pattern—for PXN, a slight decrease was observed in the microorganism content until 30 days, and a higher value was obtained at 40 days, but these variations were not considered significant. In the same way, the number of microorganisms resistant to NEMP remained almost the same during the time analyzed.

The viability of cultivable microorganisms during all the time experiments showed that there were biotic factors interfering in the fate of OPs in the natural soil samples analyzed. The maintaining of microorganism amount during the time also indicated that the ongoing biological processes did not favor growth of these kinds of living beings.

### 2.4. Methodological Analytical Parameters

The results obtained in the quantitative method evaluation are summarized in Table 4 and Table 5. As approximation, they were considered valid for both sets of soil samples sterilized and not sterilized because most of the chemical and physical properties in the samples seemingly remained equal.

### 2.5. Microbial Degradation Study

**Figure 11:**
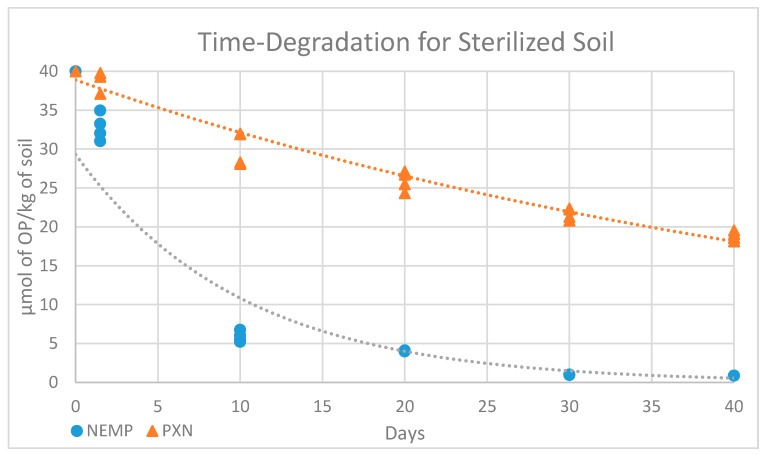
Experimental results to degradation in time of NEMP and PXN in sterile soil sample.

**Figure 12:**
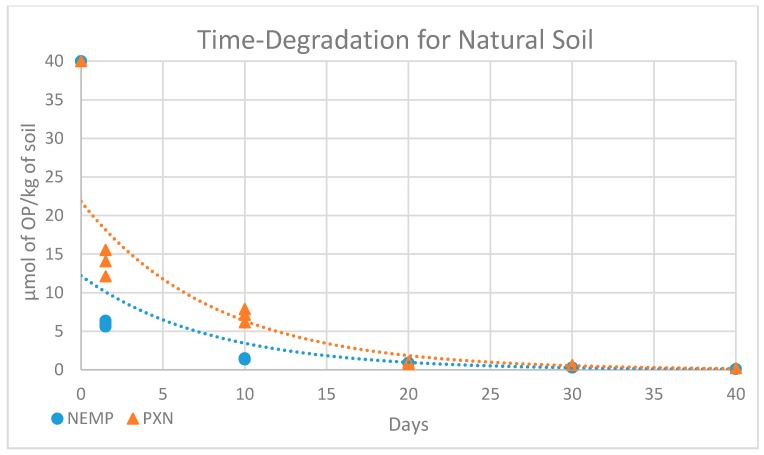
Experimental results for degradation in time of NEMP and PXN in natural soil sample.

The time-degradation observed for NEMP and PXN in sterile and non-sterile soil samples at 25°C are exhibited in Figure 11 and Figure 12 and Table 6 and Table 7. A decrease in both OP contents was observed, and the data were compared using values in micromole of OP per kilogram of soil.

The results for OP degradation in soil, in sterile or non-sterile conditions, can be better interpreted considering a first-order kinetics, as most of the time-degradation series follow this model. It was possible to obtain, through exponential regression, equations with *R*^2^ values calculated for each set of data, as summarized in Table 8.

To all tendency lines, the correlation coefficient *R*^2^ ≥ ≈0.9, showing that this kind of equation is suitable for the data obtained. The half-life time (t_1/2_) of NEMP and PXN were calculated using the equations in Table 8, considering that, at this time, the concentration y would be one-half of the initial concentration (Table 9).

Comparing data obtained for OP elimination in non-sterile and sterile soil samples, it was observed that there is a difference in the initial concentration value measured. Despite the differences in the samples, it was expected that the initial concentrations would be closer, as they were fortified at the same time with the same volume of solution of equal concentration. However, comparing the data obtained between the non-sterile and sterile samples in day 1, there was a reduction of ≈83% to NEMP and ≈65% to PXN in the content. These data show that biotic factors eliminated the OPs, making them unavailable for extraction in a very fast way, with this process starting at the primary contact between the OPs and the soil matrix.

The observed decomposition of OPs in the sterile soil samples showed that non-biological degradative mechanisms also have a contribution in the natural fate of the OPs analyzed. To better comprehend the behavior of OPs in sterile soil, it is important to understand that it depends on a variety of complex dynamic physical and chemical abiotic processes, including sorption–desorption, volatilization, and chemical degradation [6].

Using predictive models and tools for assessing chemicals under the Toxic Substances Control Act (TSCA) developed by United States Environmental Protection Agency (EPA) [32], a volatilization 22 times higher for NEMP than for PXN was estimated (Bond Structure Activity Relationships method). The sorption depended on the presence of inorganic chemicals, metals, and clay content. However, the main factor appeared to be the interactions with soil organic matter content [6]. The PXN structure was larger than NEMP due to the presence of one more oxygen atom, and thus it was expected that NEMP interacted better with empty spaces in soil organic matter, suffering in consequence a better sorption.

For chemical degradation, the main factors considered were the nature of pesticide, water content, soil constituents, and pH [33]. Water was important to consider because the solubility in this solvent determines the availability of the substance in the chemical degradation process. The predicted solubility in water (TSCA-EPA) for NEMP was four times higher than for PXN. However, it is also well known that OPs suffer hydrolysis as a chemical degradation process, and considering the soil pH (5.9, moderate) and the high content of metals (K and Ca, principally), the effects of metal ion-catalyzed hydrolysis are more probable in favoring degradation instead of the effect of pH. The metal ions can catalyze this process inducing deprotonation and metal ion coordination of water to create a reactive nucleophile. In this context, the P of PXN is more susceptible to nucleophilic attack than the P of NEMP, thanks to the presence of the O-ethyl group instead of a methyl group bonded directly to P.

The results obtained in the time elimination study revealed that for NEMP, abiotic factors contributed more than for PXN, which could be highlighted by the differences to t_½_ measured: 6.97 to 36.29, meaning that the abiotic elimination process of NEMP observed occurred more than five times faster. This result is consistent with the analysis of the principal known abiotic factors to degradation in soil, presented above.

The difference between the decomposition in non-sterile and sterile soil samples can be considered a result mainly of microbial elimination and enzymatic effects. In non-sterile samples, it was more difficult to compare the results obtained for NEMP and PXN because there were more interferences involved. However, comparing the half-life time obtained for both contaminations in natural soil, it was possible to understand that the biotic fate was more important in the elimination of PXN over NEMP, as PXN t_½_ was 5.62, almost the same as NEMP t_½_ at 5.49. The biotic contribution to NEMP can be considered smaller in relation to PXN, keeping in mind that for natural soil samples both biotic and abiotic factors work together, and that for NEMP abiotic factors are more relevant than for PXN.

To investigate the formation of the degradation products, we searched the hydrolysis products that were expected following the reactions shown in Figure 2. By comparison of mass spectra obtained at T_r_ = 14.05 (Supplementary Information, Figure S2), the presence of 4-nitrophenol was confirmed by using the Probability Based Matching (PBM) search algorithm and NIST14 library (match factor = 91). The formation of this compound was an important parameter for both OPs investigated, as this is a common product in the hydrolysis processes of NEMP and PXN.

The formation of this compound was verified in sterile and non-sterile samples as an initial degradation step. However, in non-sterile samples, a faster elimination of 4-nitrophenol and the formation of other hydrolysis products as continuation of the biodegradation steps was observed. This behavior was expected, as the enzymes and microorganisms have the capability of continuously degrading products.

Other important products monitored in the degradation process were the alkyl phosphonic acids. Their presence was characterized after the derivatization step as trimethylsilyl-derivative in both sterile and non-sterile samples, but the amount detected was higher for sterile samples. Many factors can make the detection of the alkyl phosphonic acid formed a difficult process, such as the strong interaction with metals present in the sample maintaining the alkyl phosphonic acids in salt form, enzymatic interaction and even sorption. Mass spectra obtained at T_r_ = 8.14 showed the identification of ethyl trimethylsilyl methylphosphonate, a derivative of the alkyl phosphonic acid obtained in the hydrolysis of NEMP. The data (Supplementary Information, Figure S3) were obtained using the PBM search algorithm and VGWD2019 library (match factor = 91).

The results obtained corroborates that the hydrolysis was, in fact, the principal process of degradation in both abiotic and biotic processes. However, it was favored by the presence of microorganisms once in non-sterile samples the hydrolysis process was accelerated, what shows that microbial elimination was an important factor for the metabolism of OPs in soil.

## 3. Materials and Methods

### 3.1. Molecular Docking Studies

Docking studies of PXN and NEMP were performed inside PTE from *Brevundimonas diminuta* obtained from the Protein Data Bank (PDB) (https://www.rcsb.org/) under the code 1PSC (resolution 2 Å), using the software Molegro Virtual Docker [34]. The 3D structures of the OPs were constructed using the software Spartan 08 (Wavefunction, Irvine, CA, USA) [35]. The structures were optimized and had partial charges calculated by DFT method (B3LYP, 6–31G+) and incorporated in the mol2 files used for the docking studies. In order to validate the docking methodology, a redocking procedure was performed with the ligand EBP inside the crystallographic structure of the enzyme. The ligand was constrained into a sphere of 6 Å of radius and centered at the EBP coordinates X = 31.33, Y = 12.38, and Z = 40.14. About 10 runs were performed with different parameters of population size and maximum of interactions, generating 300 poses. The best pose was chosen according to RMSD values and atom superposition criteria. After the validation, the docking studies with PXN and NEMP were performed. The OPs were constrained into a sphere of a 14 Å radius centered at the EBP, and the residues which were up to 6 Å from the OP were considered flexible. About 10 runs for each compound were performed for varying parameters of population size and maximum of interactions, and 300 poses were obtained for each OP. The best poses were chosen according to intermolecular energy and distance O(OP)-Cd. The chosen conformations were submitted to 50 ns of MD simulations in order to corroborate docking results.

### 3.2. Molecular Dynamics Studies

The enzyme and the best poses obtained from the docking studies were parameterized for the OPLS/AA forcefield [36]. Coordinates and topology files were obtained using the software AnteChamber PYthon Parcer InterfacE (ACPYPE) [37]. The MD simulations for the systems PTE/PXN and PTE/NEMP were performed using the software GROMACS 5.1.4 [38]. The systems were inserted in cubic box containing approximately 14,409 TIP4P water molecules for PXN and 16,759 for NEMP. The protocol for energy minimization included the algorithm steepest descent with position restrained (PR) of ligands and protein, steepest descent without PR to flexibilization of the systems, and conjugate gradients (CG). The minimized complexes were submitted to MD simulations in two parts. First, 500 ps of MD were performed at 310 K, with PR for the entire system, except the water molecules, to balance the solvent molecules around the enzyme. After that, 50 ns of MD were performed at 310 K without any restriction, using 2 fs of integration time and a cut-off of 10 Å for long-distance interactions. Counter ions (Cl^−^) were added to neutralize the whole system. At the end of simulations, the trajectories of all the system components were visualized on the software VMD [39]. Plots of total energy, distance, RMSD, F-RMSD, and number H-bonds formed during the MD simulation (NumligH) were generated with the programs gmx ener, gmx rms, and hbonds [38], and plotted on the Grace program (http://plasma-gate.weizmann.ac.il/Grace/).

### 3.3. Spiking Chemicals

Paraoxon-ethyl (PXN) (PESTANAL, analytical standard, 97.5% of purity) was purchased from Sigma-Aldrich (São Paulo, São Paulo, Brazil). Ethyl para-nitrophenyl methylphosphonate (NEMP) is a known surrogate for VX. It has been synthesized following the steps described by Cavalcante et al. [40] in the laboratory of organic synthesis of the Chemical, Biological, Radiological and Nuclear Defense Institute from Brazilian Army. The purity measured by NMR spectroscopy was 84% measured by 1H and 13C nuclei spectra referred to tetramethylsilane with Varian Unity 400 MHz (Palo Alto, California, USA) and Bruker Avance 400 MHz (Billerica, MA, USA). 

These compounds were chosen as they are less toxic and volatile than warfare nerve agents in general, but have a similar behavior in the environment and in contact with enzymes such as AChE and PTE. Moreover, the use and production of these substances is not prohibited by the CWC. PXN was used extensively in agricultural activities and NEMP is classified as a schedule 2.B.04 chemical in accordance with the CWC Annex on Chemicals.

### 3.4. Soil Samples

#### 3.4.1. Sampling and Initial Preparation of Soil Samples

A soil fraction was collected from the Guaratiba Environmental Reserve, located in the west zone of Rio de Janeiro, Brazil. The location of the collection point can be given by the following geographic coordinates 23.030097/43.575808 (latitude/longitude). This soil was chosen because it is a region of native soil not exploited by agricultural practices, and therefore free from previous contamination by OPs. In addition, the area has predominantly herbaceous vegetation, with grasses covering most of the land, which facilitate the removal of topsoil vegetation. The site is also close to a mangrove area that, due to its characteristics, features soil rich in nutrients, and has a notorious presence of herbivorous animals, favoring the growth of a very diverse microbiota on the site.

The collection was performed over 3 days spaced from each other over a period of 1 week to 10 days. On each occasion, five distinct points were chosen from a demarcated area containing approximately 50 m^2^. At points A, B, C, D, and E, about 100 g of soil was collected at depths between 5–10 cm, totaling approximately 500 g in each day. A total of 1500 g of soil samples were initially obtained from the region. All samples were separately packaged in a screw-capped plastic recipient previously autoclaved. In order to obtain a larger amount of microorganisms on the soil surface, including the presence of spores, it was decided that collection would take place after the occurrence of rain [41].

However, given the need for standardizing the moisture content of the samples, they were submitted to a drying protocol. This consisted of placing the samples in previously autoclaved plastic trays and subjecting the sets to 35 °C for a period of 48 h. It was considered that at this temperature, the relevant microbiota would remain unchanged. At the end of the period, the final moisture content was measured by gravimetric method as follows: different masses of soil samples were weighed and placed in a forced ventilation oven (Ecocell, Prague, Czech Republic) at 105 °C for 24 h, and the dry samples were then weighed, with the difference between the initial mass and the final mass considering the residual water mass. The percentage humidity, u (%), was calculated by dividing the residual water mass multiplied by 100 by the dry land mass [42]. The residual moisture measured presented an average close to 7%.

As an additional treatment, the total mass was then screened through 18 mesh (with 1 mm^2^ openings), eliminating residues with a particle size larger than this size, consisting mostly of stones and plant fragments. The final mass was approximately 1200 g, 800 g for experiments and 400 g for physicochemical characterization. The collected soil was kept at room temperature maintained at 25 °C by refrigeration system. To conduct the experiments, the samples were then placed in previously autoclaved screw-capped glass flasks with a capacity of 6 mL in fractions of 3 g per container. In total, 168 vials were separated, with 105 remaining unchanged and the other 63 taken for sterilization procedures.

#### 3.4.2. Analysis of the Physicochemical Characteristics

A mass of 400 g of the soil sample collected was analyzed for physicochemical characteristics at the Center of Analysis of the Federal Rural University of Rio de Janeiro. The main characteristics for soil microorganism persistence—granulometry, calcium content, organic matter, and pH—were evaluated. The textural class was defined on the basis of the triangular diagram indicated by the United States Department of Agriculture [43]. The physicochemical characteristics evaluated are summarized in Table 10 and Table 11.

#### 3.4.3. Sterilization Process

The 63 separated soil samples were sterilized following this procedure: samples were placed in an autoclave set to maintain a temperature of 121 °C for 1 h. After this time samples were removed and left at room temperature for 23 h. The entire process was repeated for 3 alternate days, with samples kept in their respective closed vials all the time. After this treatment, the samples containing the sterile soil, together with the samples containing the non-sterile soil, were stored at 4–8 °C in a refrigerator until the beginning of the experiment period.

To verify that the above protocol had been effective in promoting the sterilization of existing microbiota, including sporulated microorganisms, a sterility test was performed. Therefore, the research of aerobic and anaerobic bacterial contaminants was done in a sodium thioglycolate fluid culture medium (Difco), similarly to the practice of sterility testing of pharmaceutical products elaborated by pharmaceutical industries [44]. Four tubes with 30 mL of sodium thioglycolate fluid culture medium were heated to 90 °C to expel oxygen. When the color turned yellow, they were cooled and inoculated with the aid of a sterile spatula. Three of the sterile soil samples (3 g) were taken and aseptically all the contents of each sample were transferred to a correspondent tube. One tube without inoculum was used as a control. All tubes were incubated at 33 °C for 14 days aerobically.

After 7 and 14 days, observations were made. The sterility of the sample was confirmed by the absence of turbidity of the culture medium in the final period. Only then was the soil used for further experiments.

#### 3.4.4. Contamination with Spiking Chemicals

The 165 remaining soil samples, 105 non-sterilized and 60 sterilized, were divided in three groups of 55 samples, 35 in natural conditions and 20 sterilized in the process described above. To each sample in the first group, 120 µL of ultra-purified water was added, and to the second group the same amount of a solution of 1 mM of Paraoxon-ethyl in water, whereas to the third group a water 1 mM solution of NEMP was added. This concentration was chosen for safety reasons and to allow rigor in the measurement of sensitivity and detection limits. All the procedures were done in fume hood with controlled air flow, and all materials in the water used to prepare the solutions were sterilized.

### 3.5. Time Degradation Experiment

Each group of 55 samples with addition of water or one of the two different OP contaminations were divided into 5 groups of 11 samples, 4 sterilized and 7 not sterilized. The initial amount present (zero) was considered the value estimated of contamination (40 micromole of OP per kilogram of soil). From day 1, the first 11 samples of each group were separated to measurements, in a total of 44 samples—32 destined for chemical analysis and the other 12 to test the microbial viability. The initial measurement was made at day 1, once time was needed to prepare all samples and analyze the first ones. As a result, the effects of sample soil matrix in the analytes during this period needed to be taken into account. The other samples stayed in a room with the temperature maintained at approximately 25 °C (minima at 23 °C and maxima at 27 °C, measured during all the experimentation time) and were each separated at four time intervals: 10, 20, 30, and 40 days. After that, the soil samples were maintained in a frosted state at −60 °C until the moment of analysis.

### 3.6. Microbial Viability in Soil

At each time interval, a set of nine samples, divided in three groups (water, PXN, and NEMP contamination) were evaluated. The microbial viability study was carried out in triplicate using a plate-counting procedure as follows: the first step was the homogenization of the soil samples performed manually with rotational movements in horizontal and vertical axes for 30 min. Then, 1 g of each 3 g sample was removed and transferred to the same Erlenmeyer flask containing 300 mL of a sterile 0.1% (*w*/*v*) sodium pyrophosphate solution. Homogenization was performed in a shaker for 30 min at 33 °C and 150 rpm. The sample solution was diluted into a 10-fold series up to 10^−5^.

From each serial dilution, 0.1 mL aliquots were removed and spread with a Digralski spatula on tryptic soy agar (TSA) culture medium plate surface autoclaved at 121 °C for 30 min and cooled prior to use. This media was chosen because in the test performed by Vieira and Nahas [45], for quantification of total bacteria in soil samples, it presented the best results. This procedure was replicated three times in different plates.

Aerobic incubation was performed at 33 °C in a 24 h period, and the numbers of colonies that appeared on the plates after that were counted. The dilution, which had 30–300 microorganisms, was selected as a standard. To determine the total cells in each type of sample, a set of nine datum obtained (three counts from three plates) was used, and the mean value and standard deviation were measured. The number of microorganisms in the sample per gram of soil was calculated as shown in Equation (1). The 10^3^ constant was included to adjust the value obtained proportionally to 1 g of soil, as each 1 g was diluted in 100 mL and only 0.1 mL of this solution was used in plate as described above.

(1) $$\frac{Microbial\;number}{g\;of\;soil}=\frac{10^{3}\times Average\;of\;colonies\;in\;standard\;dilution\;plate}{Dilution\;Factor}$$

### 3.7. Gas Chromatography and Mass Spectrometry Analyses

The general analysis procedure followed what has been described to verify the fate of VX and related products in forced contaminated soil samples submitted to different conditions during a determined period [46,47]. Some adaptations are necessary to have better results with spiking chemicals chosen, and for adequate methodology of materials and equipment available in the structure used; however, the main aspects for assurance of quality in the determinations made are verified [48].

#### 3.7.1. Soil Sample Preparation for Analysis

The soil samples were defrosted, extracted with ethanol (3 mL), mixed using vortex for 15 s, refrigerated at 4 °C, and centrifuged at 3600× *g* for 15 min. A total of 1.5 mL of the upper liquid was separated, filtered using a 0.45 mm nylon filter, concentrated to dryness, subsequently re-suspended in 150 µL of ACN, and agitated again via vortex for 10 s. This aliquot was analyzed by GC-MS for quantitative analysis of the degradation of OPs. After that, the same aliquot was recapped and derivatized with an addition of 45 µL of BSTFA and reaction at 60 °C for 30 min to be analyzed again in GC-MS for qualitative analysis of the degradation products.

#### 3.7.2. Instrumentation and Analysis

To analyze the OPs and their degradation products gas chromatography (GC, Agilent 6890 system, Santa Clara, CA, USA) was used, combined with mass spectrometry (MS, Agilent 5975C detector, Santa Clara, CA, USA) equipped with an autosampler. GC-MS was chosen because these combined techniques are the main way to detect and identify chemicals for verification of compliance to the CWC and for confirming CWA use in all reference laboratories worldwide [49]. The GC was performed in splitless mode with the injection port kept at 220 °C. The initial temperature of the GC oven (40 °C) was held for 1 min and ramped up 10 °C/min to 300 °C, where it was held for 10 min. An Agilent Technologies column HP 5ms Ultra Inert (30 m in length, diameter of 0.25 mm, and a film thickness of 0.25 mm) was used in constant flow mode with a flow of 1.3 mL/min helium. The total run time was 37 min and the injection volume was 1 µL. The MS data were obtained in selective ion monitoring (ions 79 (quantifier), 217 and 245 (qualifiers) for NEMP and 109 (quantifier), 149 and 275 (qualifiers) for PXN) for better quantification, and in scan mode (40–400 window) for qualitative search of degradation products.

#### 3.7.3. Internal Verification for the Methodology of Analysis

To certify that the methodology applied was efficient to quantify elimination of the compounds of interest in the soil matrix during the time period of experimentation, the following analytic parameters were evaluated: selectivity, linearity and variation range, precision, accuracy, detection, and quantification limits. The selectivity was verified comparing a soil with analytes and without it to see if there were interferences in the matrix that compromised the analysis method. Method linearity was assessed by linear regression analysis for the response obtained at five different concentrations of OP compounds repeated three times using external standardization. The variation range was maintained between 0 and 100% of the initial contamination. The precision was measured by repeatability using nine determinations (three levels with three repetitions each) and calculating the relative standard deviation obtained. The accuracy was evaluated by recovery test in triplicate of samples obtained fortifying a soil blank with three different known concentrations of the analytes of interest and performing all procedures of sample preparation and analysis. The results obtained were compared with the analysis of the solution containing PXN and NEMP in the same concentrations. Detection limit and quantification limit were measured by the method based on analytical curve parameters. All these procedures were done using natural soil and are also considered valid as an approximation to sterile soil, as apparently the differences between samples are only the biotic characteristics and thus the experimental cost was not justified in performing validation procedures for both set of samples.

#### 3.7.4. Data Analysis

Initial data analysis was performed using MassHunter (Agilent Technologies, Santa Clara, CA, USA). Data were exported to Microsoft Excel for quantification and graphical representation. Quantification was based upon the peak area obtained with automatic integration in the chromatogram selective ion monitoring of the following ions and time retentions (tr) windows: *m*/*z* = 109 and tr = 18.3 ± 1 for Paraoxon-ethyl, and *m*/*z* = 79 and tr = 17.4 ± 1 for NEMP. Concentration results were obtained for the different sample preparations and analyses of the three soils, which were injected in duplicate. The results were adjusted to achieve the concentration present in micromole of OP per kilogram of soil samples considering all procedures performed and the methodology evaluation parameters.

## 4. Conclusions

In this work, molecular modeling and experimental studies were performed to investigate the elimination process of PXN and NEMP. The experimental results showed that biotic processes played an important role in the degradation process in soil samples. Hydrolysis can be confirmed as the principal transformation mechanism because of the formation of characteristic products that were detected in the analytical tests. For NEMP, the biotic factors were less important in the elimination process than for PXN. Using the enzyme PTE, we obtained theoretical results that suggested that PXN keeps closer to the active site than NEMP. These results also showed that PXN had lower intermolecular interaction energy and that it kept closer to the active site, favoring hydrolysis. NEMP was kept away from the active site, which could even indicate an inhibition of the enzyme on an allosteric site. This can be toxic to microorganisms instead of favoring elimination in time. The theoretical and experimental results compared together suggest that protocols involving studies with key enzymes can contribute to investigate and maybe predict the biodegradation process observed in natural environments. Additionally, it can be used to better understand the fate of other OPs, including chemical warfare agents and new pesticides.

## Figures and Tables

**Table 1:** Docking results for the organophosphorus (OP).

OP	Distance O(OP)-Cd (Å)	Distance O(Asp301)-Cd	Intermolecular Energy (kcal/mol)	H-Bond Energy (kcal/mol)	H-Bond Interaction Residues
PXN	3.57	3.21	−99.33	−0.26	Trp131
NEMP	4.56	3.21	−89.40	−0.69	His257

**Table 2:** H-bond interactions for the systems PTE/PXN and PTE/NEMP.

OP	Average H-Bond Number	Interaction Residues
PXN	1	Asn312
NEMP	1	Ser75
		Lys82
		Ser267
		Phe306
		Ser308
		Tyr309
		Thr311
		Asn312

**Table 3:** Microbial viability of soil.

Sample	Soil with Water	Soil with PXN	Soil with NEMP
0 days	$\;7.6\pm 0.1\times 10^{9}\;$	$\;5.4\pm 0.1\times 10^{8}\;$	$\;8.4\pm 0.2\times 10^{7}\;$
10 days	$\;7.5\pm 0.1\times 10^{9}\;$	$\;12.2\pm 0.2\times 10^{7}\;$	$\;8.1\pm 0.3\times 10^{7}\;$
20 days	$\;7.9\pm 0.5\times 10^{9}\;$	$\;8.2\pm 0.2\times 10^{7}\;$	$\;7.3\pm 0.2\times 10^{7}\;$
30 days	$\;8.1\pm 0.4\times 10^{9}\;$	$\;7.8\pm 0.3\times 10^{7}\;$	$\;8.0\pm 0.3\times 10^{7}\;$
40 days	$\;7.5\pm 0.3\times 10^{9}\;$	$\;11.4\pm 0.5\times 10^{7}\;$	$\;7.6\pm 0.1\times 10^{7}\;$

**Table 4:** Evaluation of the quantitative methodology results.

Parameter	Criteria	NEMP	PXN
Selectivity	Not find any interference in the analysis of soil blank.	No interference at retention time 17.4 ± 1.	No interference at retention time 18.3 ± 1
Linearity	Linear regression; *R*^2^ values above 0.9 was considered satisfactory. Variation range: 0 to 40 µmol/kg. Points: 0, 8, 16, 24, 32, 40 µmol/kg.	y = 157,640.29 x − 413,553.34 *R*^2^ = 0.92 (satisfactory)	y = 847,053.87 x − 1592,824.70 *R*^2^ = 0.94 (satisfactory)
Precision	Repeatability measured by relative standard deviation; mean value below 20% was considered satisfactory. Levels: 8, 24, 40 µmol/kg.	15.81 ± 5.57% (high, satisfactory)	16.28 ± 5.61% (high, satisfactory)
Detection limit	Method based on analytical curve parameters; $DL=3.3\times \frac{s}{S}$; *s* = standard deviation of linear coeficiente of equation and S = angular coeficient.	0.86 µmol/kg of soil	0.62 µmol/kg of soil
Quantification limit	$\;QL=10\times \frac{s}{S}\;$.	2.90 µmol/kg of soil	2.07 µmol/kg of soil

**Table 5:** Accuracy and recovery test results.

Accuracy						
OP	Concentration (µmol/kg)			Mean Value + SD	Criteria	Aceptance
	8	24	40			
Recovery for NEMP	42.37%	46.45%	53.14%	47.32 ± 5.44%	Recovery test; mean value for all variation range above or near to 50% was considered satisfactory	Satisfactory
Recovery for PXN	41.30%	59.59%	69.95%	56.95 ± 14.50%		Satisfactory

**Table 6:** Values with intervals for 95% of confidence obtained in sterile soil (µmol of OP/kg of soil).

	0	1 Day	10 Days	20 Days	30 Days	40 Days
NEMP	40	32.82 ± 1.8	5.86 ± 0.73	4.04 ± 0.05	0.98 ± 0.03	0.86
PXN	40	39.4 ± 1.90	30.06 ± 2.31	25.91 ± 1.35	21.66 ± 0.80	18.83 ± 0.65

**Table 7:** Values with intervals for 95% of confidence obtained in natural soil (µmol of OP/kg of soil).

	0	1 Day	10 Days	20 Days	30 Days	40 Days
NEMP	40	6.02 ± 0.34	1.44 ± 0.07	0.89 ± 0.01	0.86	0.86
PXN	40	13.45 ± 1.77	6.82 ± 0.90	0.85 ± 0.13	0.62	0.62

**Table 8:** Degradation experiment equations for NEMP and PXN.

	NEMP		PXN	
	Equation	*R*^2^ Value	Equation	*R*^2^ Value
Natural soil	$\;y\;=\;12.21e^{-0.13x}\;$	0.89	$\;y\;=21.82e^{-0.12x}\;$	0.94
Sterilized soil	$\;y\;=\;29.3e^{-0.10x}\;$	0.93	$\;y\;=\;38.89e^{-0.02x}\;$	0.96

**Table 9:** Half-life for NEMP and PXN.

NEMP t_1/2_-Value		PXN t_1/2_-Value	
Natural Soil	Sterilized Soil	Natural Soil	Sterilized Soil
5.49	6.97	5.62	36.29

**Table 10:** Physicochemical characteristics of soil samples.

pH (In Water)	P	K	Ca	Mg	Al	H + Al	Na
5.9 (moderate)	69 mg/dm^3^	72 mg/dm^3^	2.8 cmol_c_/dm^3^ (high)	0.8 cmol_c_/dm^3^	0.0 cmol_c_/dm^3^	2.2 cmol_c_/dm^3^	0.1 cmol_c_/dm^3^

**Table 11:** Physicochemical characteristics of soil samples (continuation).

N	C	Electric Conditivity	Cation Exchange Capacity		Organic Matter	Granulometry		Class/Texture
**0.29%**	**1.99%**	0.21 dS/cm	Effective	3.9 cmol_c_/dm^3^	34.3 g/dm^3^ (high)	Sand	778 g/kg	Sandy
			pH 7	6.1 cmol_c_/dm^3^		Silt	156 g/kg	
			Sum of bases	3.9 cmol_c_/dm^3^		Clay	66 g/kg	

## Supplementary Materials

The following are available online at https://www.mdpi.com/1420-3049/25/1/58/s1, **Figure S-1.** Mass Spectra comparison for 4-nitrophenol; **Figure S-2.** Mass Spectra comparison for Ethyl trimethylsilyl methylphosphonate; **Figure S-3.** RMSD plots ligand/protein for PXN and NEMP during 50 ns of MD simulation.
