# Supplementary material for: Biodegradation of Organophosphorus Compounds Predicted by Enzymatic Process Using Molecular Modelling and Observed in Soil Samples Through Analytical Techniques and Microbiological Analysis: A Comparison
Source: Molecules. 2019 Dec 23;25(1):58. doi: 10.3390/molecules25010058 (PMC6982719; doi:10.3390/molecules25010058)
Supplement: Supplementary file 1 [file molecules-25-00058-s001.pdf]

molecules

MDPI

Article

Biodegradation of organophosphorus compounds predicted by enzymatic process using molecular modelling and observed in soil samples through analytical techniques and microbiological analysis: a comparison.

Monique Cardozo [1,2,*], Joyce S. F. D. de Almeida [3], Samir F. de A. Cavalcante [1,2,4], Jacqueline R. S. Salgado[2], Arlan S. Gonçalves[5,6], Tanos C. C. França [3,4], Kamil Kuca [4,*] and Humberto R. Bizzo [1]

1 Natural Products Research Institute (IPPN), Federal University of Rio de Janeiro (UFRJ), CCS, Bloco H, Cidade Universitária, Rio de Janeiro 21941-902, Brazil; samirfac@yahoo.com.br (S.F.d.A.C.); humberto.bizzo@embrapa.br (H.R.B.)
2 Institute of CBRN Defense (IDQBRN), Avenida das Américas 28705, Rio de Janeiro 23020-470, Brazil; capjrsoares@gmail.com (J.R.S.S.)
3 Laboratory of Molecular Modeling Applied to Chemical and Biological Defense (LMACBD) Military Institute of Engineering (IME), Praça General Tibúrcio 80, Rio de Janeiro 22290-270, Brazil; joycesfdalmeida@gmail.com (J.S.F.D.d.A.); tanosfranca@gmail.com (T.C.C.F.)
4 Department of Chemistry, Faculty of Science, University of Hradec Kralove, Hradec Kralove, 50003 Post code, Czech Republic.
5 Federal Institute of Education, Science and Technology, Avenida Ministro Salgado Filho, 1000, Soteco, Vila Velha 29106-010, Espírito Santo, Brazil; arlansgoncalves@gmail.com (A.S.G)
6 Federal University of Espirito Santo- Unit Goiabeiras, Vitória 29075-910, Espírito Santo, Brazil
* Correspondence: niquecard@gmail.com (M.C.); kamil.kuca@uhk.cz (K.K.); Tel.: +55-021-2410-6305 (M.C.)

Academic Editors: Pascal Houzé and Frédéric J. Baud

Received: 1 November 2019; Accepted: 19 December 2019; Published: 23 December 2019

Supplementary Information

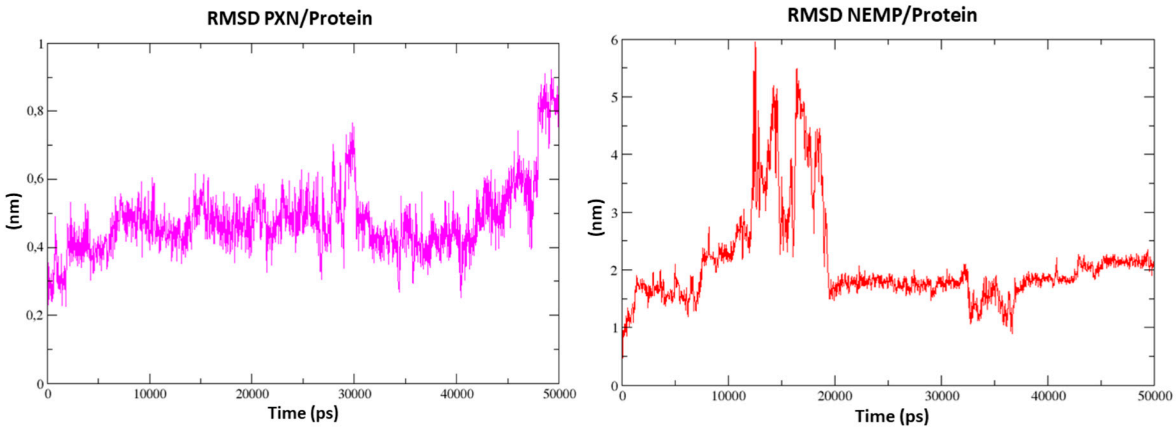

Figure S1. RMSD plots ligand/protein for PXN and NEMP during 50 ns of MD simulation.

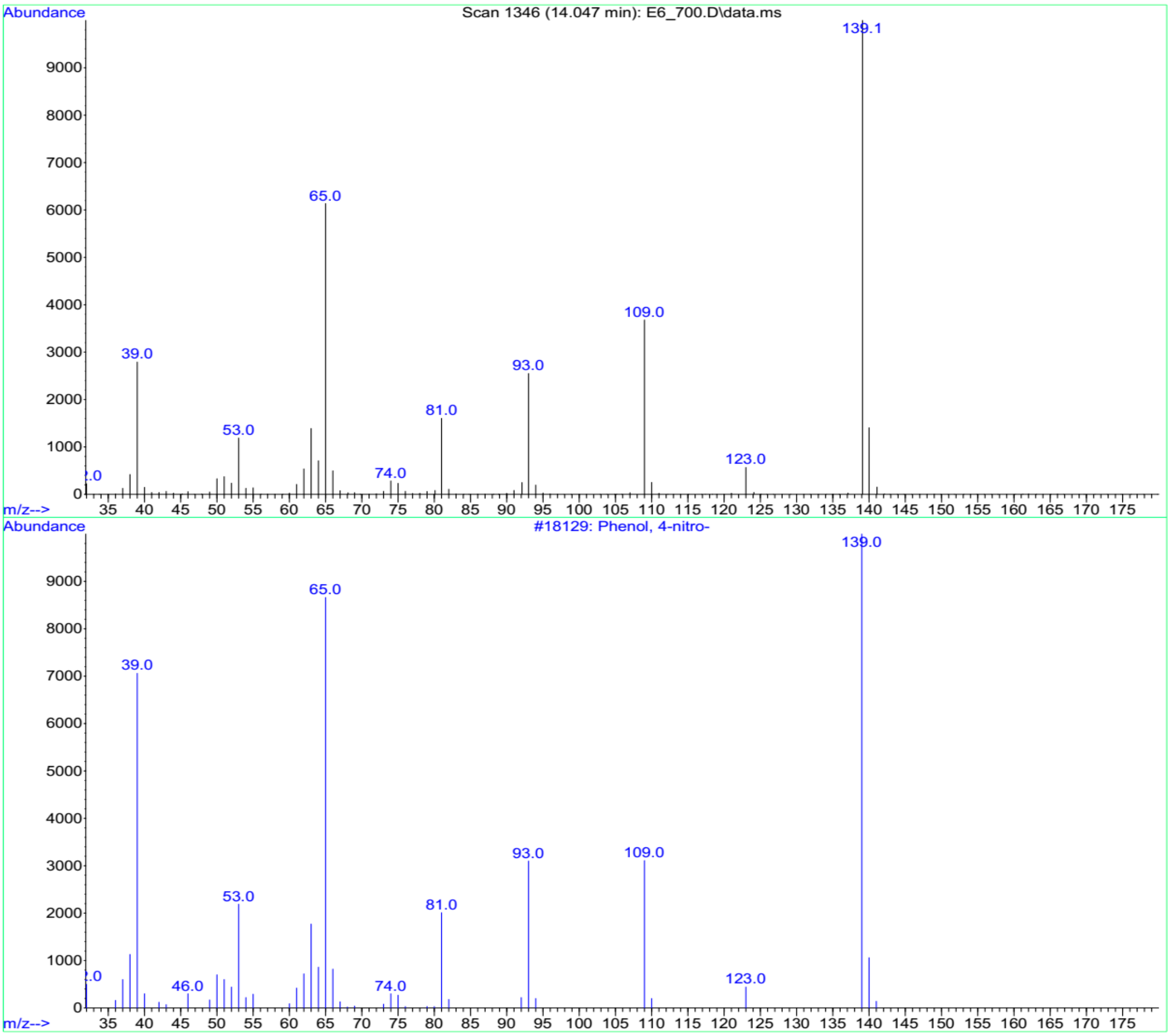

Figure S2. Mass Spectra comparison for 4-nitrophenol.

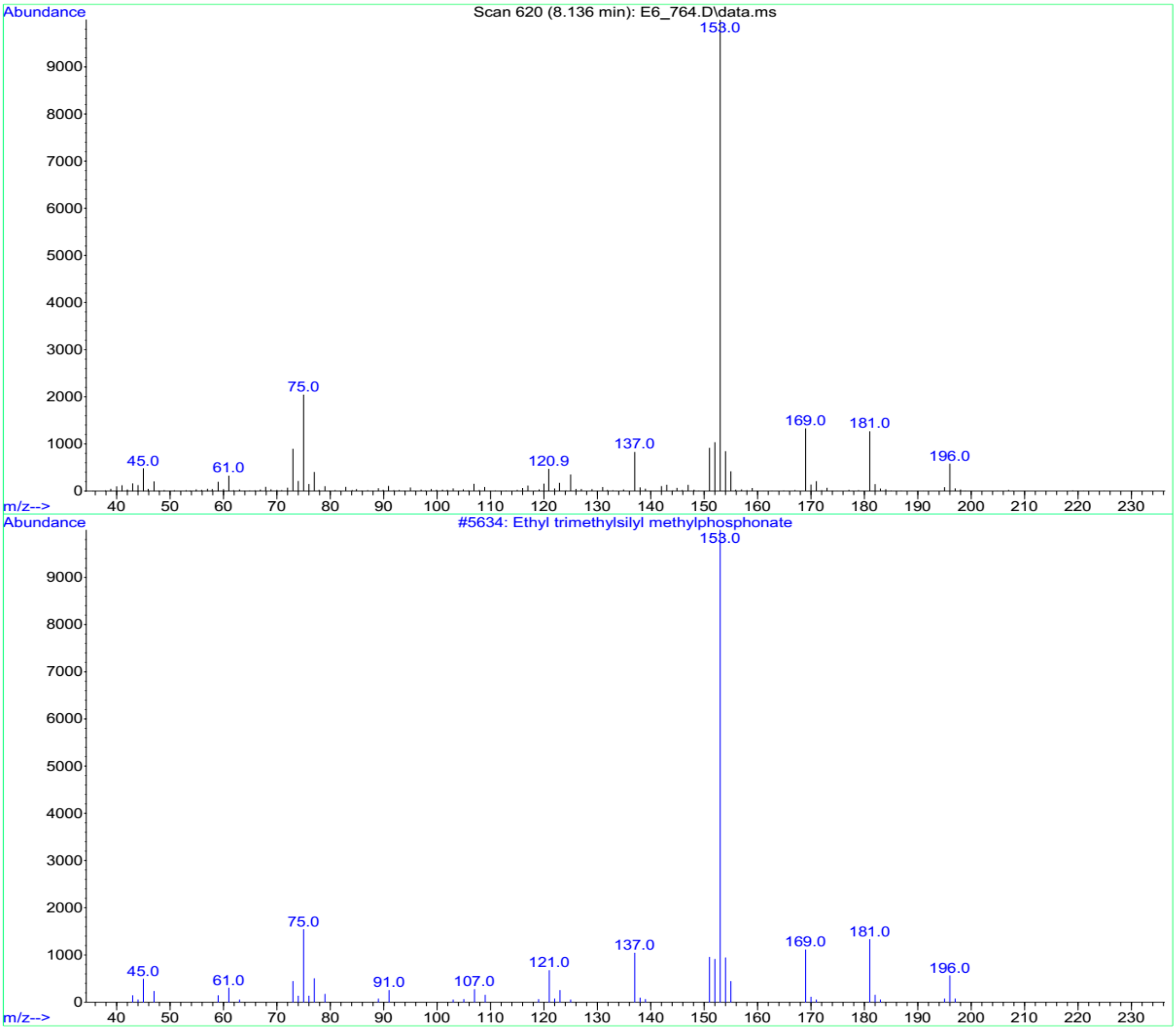

Figure S3. Mass Spectra comparison for Ethyl trimethylsilyl methylphosphonate.
